# Exploring Genetic Polymorphism of *Plasmodium vivax* Variant Interspersed Repeats (*vir*) 12, Subfamily E, in Clinical Samples From Thailand

**DOI:** 10.1155/jotm/5898346

**Published:** 2026-10-21

**Authors:** Jiraporn Kuesap, Nutnicha Suphakhonchuwong

**Affiliations:** ^1^ Faculty of Allied Health Sciences Thammasat University Pathumthani 12120 Thailand tu.ac.th; ^2^ Faculty of Medicine Praboromarajchanok Institute Nonthaburi 11000 Thailand

**Keywords:** genetic polymorphism, malaria, *Plasmodium vivax*, *Plasmodium vivax* variant interspersed repeats, vir

## Abstract

The *Plasmodium vivax* variant interspersed repeat (*vir*) gene family is the largest multigene family in human malaria parasites and belongs to the *pir* superfamily. These genes encode variant surface antigens that are thought to contribute to immune evasion and disease pathogenesis. Among them, *vir* 12 of subfamily E is relatively conserved but exhibits polymorphism that may provide useful insights into the genetic diversity of *P. vivax*. A total of 100 *P. vivax* DNA samples were collected from finger‐prick blood specimens in Yala province, Thailand, between 2017 and 2019. The *vir* 12 gene was amplified and sequenced, and analyses were conducted to assess genetic diversity and neutrality tests. The *vir* 12 gene exhibited low genetic diversity (*π* = 0.00016) among studied isolates with 6 haplotypes. Neutrality tests indicated evidence of purifying selection, suggesting immune‐driven evolutionary pressures. Comparative analysis with other geographic populations revealed both shared and region‐specific alleles, highlighting localized transmission dynamics and underlying parasite population structure. This study demonstrates low genetic polymorphism of *vir* 12 in *P. vivax* populations from Yala province, Thailand, consistent with reports from other regions but with distinct patterns across endemic areas. These findings underscore the importance of investigating conserved members of the *vir* family to enhance our understanding of parasite adaptation and host–parasite interactions, with implications for malaria control and vaccine development.

## 1. Introduction

Malaria remains a significant mosquito‐borne disease of humans, resulting from infection with protozoan parasites of the genus *Plasmodium*. *Plasmodium vivax* is responsible for the majority of malaria infections in Central and South America, Central Asia, South Asia, Southeast Asia, Middle East, Oceania, and East Africa [1] and remains a major public health challenge because of its ability to form dormant hypnozoites, cause recurrent infections, and persist at low peripheral parasitemia that evades routine detection and control measures [2]. Although *P. vivax* is generally considered nonfatal, severe vivax malaria has been linked to complications such as severe anemia, respiratory distress, lung and splenic injury, renal failure, unconsciousness, low birth weight, and malnutrition [3, 4].

Genomic investigations have shown that *P. vivax* exhibits greater genetic diversity than *P. falciparum*, which may complicate vaccine development and facilitate immune evasion [5–7]. Malaria parasites evade host immunity through antigenic variation of surface antigens (VSAs), a process that can worsen disease by delaying immune recognition and promoting cytoadherence and resetting [8, 9]. The VSAs proteins are encoded by multigene families situated in the telomeric and subtelomeric regions of the parasite chromosomes. Among these genes, the variant interspersed repeats (*vir*) superfamily of *P. vivax* represents the largest subtelomeric multigene superfamily, 346 genes in 12 subfamilies (A‐L) were grouped in *P. vivax* Sal‐I genome [10], 1074 genes in *P. vivax* isolate (PvPAM) [11], 1145 genes in the PvW1 genome [12], and over 1200 genes in 27 subfamilies in patient isolates (PvP01) [13]. The VIR proteins have been implicated in host immune system evasion [14] and are exported to the surface of infected erythrocytes, where partially induce the adherence of *P. vivax*‐infected reticulocytes to the endothelial cell receptor [15, 16]. The virulent nature of VIR proteins suggests their role in malaria pathogenesis, highlighting the importance of investigating their genetic diversity to better understand the evolving trends of *P. vivax*.

Currently, the genetic polymorphisms of *vir* genes of *P. vivax* isolates have been investigated in some geographical regions, including India, Korea, Myanmar, and Pakistan [17–21]. However, information regarding the genetic diversity of the *vir* genes in Thai isolates remains limited. Overall, studies on the global genetic diversity and population structure of *vir* genes are still scarce, highlighting the need for further investigations across diverse *P. vivax* populations to better understand their functional roles and potential applications in malaria control. Therefore, the objective of this study was to investigate the genetic diversity and natural selection of *vir*12, a member of subfamily E of the *vir* gene family, which exhibits both sequence conservation and high genetic diversity [17, 18], in *P. vivax* clinical isolates from Yala province, Thailand.

## 2. Materials and Methods

### 2.1. Sample Collection

A total of 100 *P. vivax* DNA samples were obtained from finger‐prick blood collected on filter paper in Yala province, southern Thailand, between 2017 and 2019. The samples were transported to the laboratory in Pathumthani for DNA extraction at room temperature, and the extracted DNA was stored at −20°C. The study protocol was reviewed and approved by the Human Research Ethics Committee of Thammasat University (Science), Thailand (COA No. 021/2566).

### 2.2. PCR Amplification

The *vir* 12 gene was amplified by seminested PCR using the previously described specific primers [17, 19]. The primary PCR primers amplified a region of 2038 bp, and seminested primers amplified a region of 1323 bp. PCR was performed using a reaction mixture containing 1.0 μM of each primer, 1 × MyTaq Reaction Buffer (Bioline, London, United Kingdom), 0.5 unit of MyTaq HS DNA Polymerase (Bioline, London, United Kingdom), and 1 μL of genomic DNA or the amplicon from the primary PCR. The primary PCR conditions were 95°C for 5 min, followed by 35 cycles of 95°C for 30 s, 50°C for 30 s, and 72°C for 1 min, with a final extension at 72°C for 5 min. The seminested PCR had 30 cycles of the annealing step. PCR products were analyzed on a 1.2% agarose gel, and band sizes were visualized under UV illumination.

### 2.3. PCR Product Purification and DNA Sequencing

Amplified PCR products were purified using QIAquick PCR Purification Kit (Qiagen, Germany). All positive samples were sequenced bidirectionally, forward and reverse, using ABI 3730XL DNA Analyzer (Thermo scientific, MA, USA). After removal of low‐quality and nonspecific bases, all successfully sequenced isolates yielded sequences of 969 bp, which were used for downstream analyses. The nucleotide sequences were submitted to GenBank databases (GenBank accession numbers: PX696666–PX696730).

### 2.4. Sequence Diversity and Neutrality Analysis

The nucleotide sequences of *vir* 12 genes were aligned and analyzed using ClustalX in MEGA X (https://www.megasoftware.net/index.html) program. Sequences were compared with Salvador I (Sal‐I) reference strain (NC_009915.1) available in GenBank. Segregating sites (S), number of haplotypes (H), the average number of nucleotide differences (K), haplotype diversity (Hd), and nucleotide diversity (*π*) among study sequences were determined using DnaSP Version 6.0 (https://www.ub.edu/dnasp/). The neutrality tests (Tajima’s D test, Fu and Li’s *D*∗ test, and Fu and Li’s F∗ test) were analyzed using DnaSP. For interpretation, under neutrality, Tajima’s D is expected to be zero. Significantly positive values interpreted as balancing selection or a population bottleneck, while negative values indicate population expansion or directional selection [22]. The phylogenetic trees were constructed by MEGA X using minimum evolution methods.

### 2.5. Haplotype Networks and Global Genetic Diversity

The genetic relationship of the *vir* 12 haplotype among the present study and previously reported [17–21] was analyzed by haplotype networks using PopArt (https://popart.maths.otago.ac.nz/) based on the median‐joining method [23]. The global nucleotide sequences were retrieved from GenBank (Table S1) including those from India, Korea, Myanmar, and Pakistan [17–21]. Additionally, the genetic diversity between populations was calculated by measuring the fixation index (*F*_ST_) using Arlequin ver. 3.1. High genetic differentiation is indicated by *F*_ST_ > 0.15 [24]. The recombination parameter (R), which included the effective population size and probability of recombination between adjacent nucleotides per generation (Ra, Rb), and the minimum number of recombination events (Rm) were analyzed using DnaSP (Version 6.0).

## 3. Results

### 3.1. Genetic Diversity and Natural Selection of *vir* 12

Among 100 *P. vivax* samples analyzed, the *vir* 12 gene was successfully amplified in 65 samples (65%), including 32/35 samples from 2017, 20/32 samples from 2018, and 13/33 samples from 2019. Comparison of the genetic sequences of the study isolates with the Sal‐I reference strain identified 73 single‐nucleotide polymorphisms (SNPs) sites. The genetic diversity among study isolates revealed 5 segregating sites (S), 6 distinct haplotypes (H), 0.15385 average number of nucleotide differences (K), 0.14904 haplotype diversity (Hd), and 0.00016 nucleotide diversity (*π*) (Table 1). Neutrality tests showed negative Tajima’s D values (−1.96374) with significant deviation from neutrality (*p* < 0.01). The Fu and Li’s *D*∗ and F∗ values were consistent with Tajima’s D values. The significant difference of Fu and Li’s *D*∗ and F∗ values was found with the value of −3.94143 (*p* < 0.02) and −3.88616 (*p* < 0.02), respectively. Genetic diversity was observed among isolates collected in 2019, whereas the same single haplotype (H) was consistently identified in isolates from both 2017 and 2018, suggesting possible clonal transmission during that period. Low recombination parameters were found (Rm: 0), indicating low meiotic recombination and thus minimal genetic diversity for this gene in population.

**TABLE 1:** Nucleotide diversity and neutrality tests of *vir* 12 in *P. vivax* clinical isolates from Thailand.

Sample years	Frequency (%)	S	H	K	Hd	*π*	Tajima’s D (*p* value)	Fu and Li’s *D*∗ (*p* value)	Fu and Li’s F∗ (*p* value)
2017	32 (49.2)	0	1	0	0	0	0	0	0
2018	20 (30.8)	0	1	0	0	0	0	0	0
2019	13 (20.0)	5	6	0.76923	0.64103	0.00079	−1.86311 (*p* < 0.05)	−2.32348 (*p* < 0.05)	−2.50594 (*p* < 0.05)
Total	65 (100.0)	5	6	0.15385	0.14904	0.00016	−1.96374 (*p* < 0.01)	−3.94143 (*p* < 0.02)	−3.88616 (*p* < 0.02)

*Note:* Total represents all successfully amplified isolates collected during 2017–2019 combined; year‐specific values represent analyses performed separately for each year.

Phylogenetic analysis revealed two distinct clades within the study population (Figure 1), with one clade comprising a single haplotype (H2) and the other containing the remaining haplotypes. No particular pattern was observed in haplotype distribution, while the Sal‐I reference strain positioned on a separate branch. Given the low genetic diversity observed, the phylogenetic analysis primarily illustrates the genetic relationships among the identified haplotypes.

**FIGURE 1:**
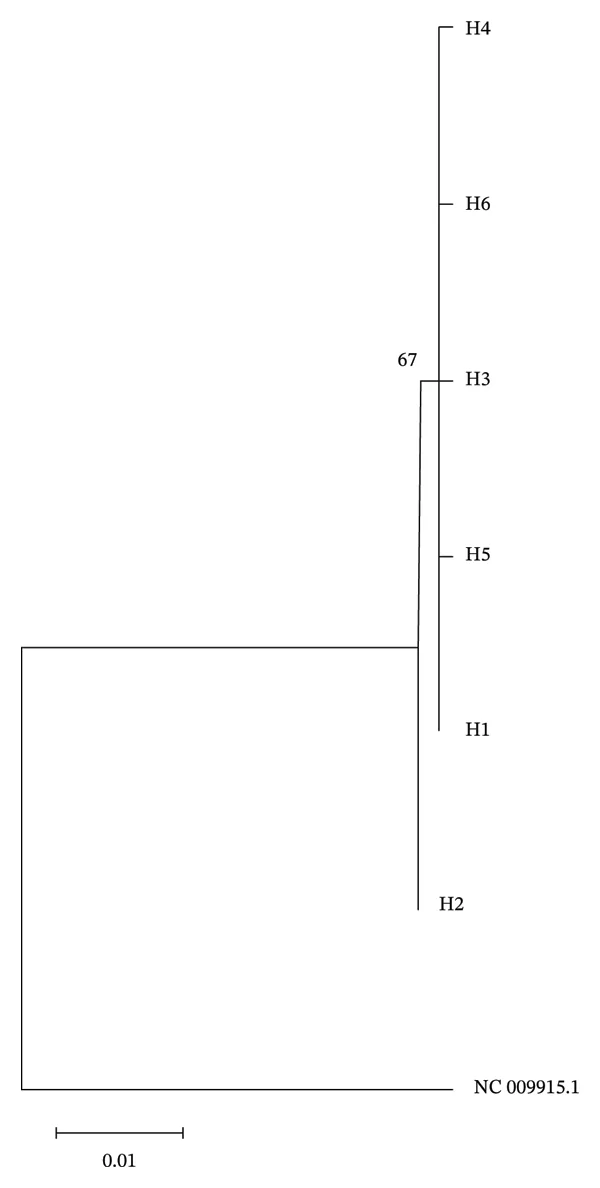
The phylogenetic tree of *vir* 12 gene.

### 3.2. The Asian *vir* 12 Haplotype Network

The haplotype networks of *vir* 12 sequences are shown in Figure 2. Forty haplotypes were identified in 157 *vir* 12 sequences from 5 countries (India, Korea, Myanmar, Pakistan, and Thailand). Haplotype prevalence ranged from 0.6% to 38.2% with high frequency of Hap_35 (60/157, 38.2%) from Thai sequences, followed by Hap_13 (13/157, 8.3%) from Korean sequences. Twenty‐eight haplotypes were identified as singleton (28/40, 70.0%), and the frequency was high in Myanmar sequences (11/28, 39.3%) followed by Indian (9/28, 32.1%), and Thai (5/28, 17.9%) sequences. The haplotype networks showed geographic clustering. Interestingly, Thai haplotypes were connected to Indian populations, while Indian populations were connected to Korean, Myanmar, and Pakistan populations. Among 40 haplotypes, Hap_1 and Hap_9 were detected in two populations.

**FIGURE 2:**
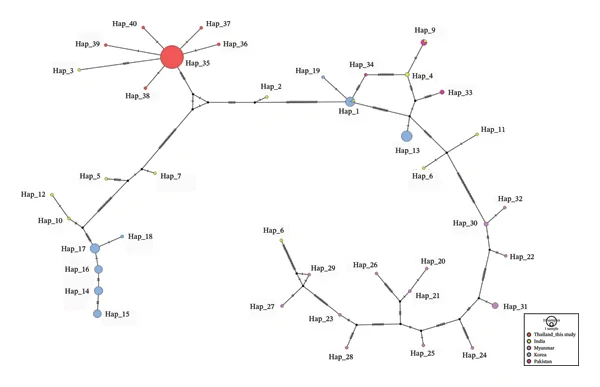
Haplotype network of Asian *vir* 12. Branch lengths are proportional to genetic divergence, and node sizes reflect total haplotype frequencies. The network includes 40 haplotypes identified among 157 sequences, with each color representing a distinct geographical origin. Lines connecting haplotypes indicate mutational steps.

The genetic diversity between Asian populations was investigated. The *F*_ST_ values are presented in Table 2. Pairwise comparisons between present study population and isolates from India, Korea, Myanmar, and Pakistan revealed high *F*_ST_ values. Likewise, comparisons between other countries also revealed high differentiation. All pairwise comparisons were statistically significant (*p* < 0.01). The high *F*_ST_ values among regions indicate substantial genetic differentiation of *vir* 12 between populations.

**TABLE 2:** Genetic diversity of *vir* 12 from five populations by pairwise *F*_ST_ values (*p* value).

	Thailand	India	Korea	Myanmar	Pakistan
Thailand					
India	0.46420 (< 0.01)				
Korea	0.65618 (< 0.01)	0.19676 (< 0.01)			
Myanmar	0.92433 (< 0.01)	0.56592 (< 0.01)	0.69402 (< 0.01)		
Pakistan	0.80478 (< 0.01)	0.19203 (< 0.01)	0.40044 (< 0.01)	0.87488 (< 0.01)	

## 4. Discussion

Malaria is a mosquito‐transmitted disease that remains a major public health concern in tropical and subtropical regions. Among human malaria parasites, *P*. *vivax* is the most widespread species, particularly in Central and South America, Central Asia, South Asia, Southeast Asia, Middle East, Oceania, and East Africa [1]. Despite its broad distribution and clinical importance, information regarding the genetic diversity of *P. vivax vir* genes in Thai isolates remains limited. Therefore, this study aimed to investigate the genetic diversity and natural selection of *vir* 12 in *P. vivax* clinical isolates from Yala province, Thailand.

The *vir* family represents the largest multigene family in human malaria parasites and belongs to superfamily *pir* (*Plasmodium* interspersed repeats) [25]. Among the currently characterized *vir* gene family, 4 *vir* genes (*vir* 4, *vir* 12, *vir* 21, and *vir* 27) have been most extensively studied [17–21], revealing varying levels of genetic polymorphism.

In the current study, the genetic diversity and evolutionary dynamics of *vir* 12, a member of subfamily E of the *vir* gene superfamily, in *P. vivax* isolates collected from southern Thailand were analyzed. The amplification success of *vir* 12 observed in this study (65%) is consistent with previous reports from Myanmar (72.5%, 29/40) [20] and Korea (69.4%, 59/85) [19], but higher than those from India (25.7%, 19/74) [17] and Pakistan (6.7%, 6/89) [21]. Differences in amplification success may be associated with sequence variation within or near the primer‐binding regions. Therefore, the exclusion of nonamplified samples may have introduced some amplification bias and potentially affected the estimates of *vir* 12 genetic diversity. A total of 73 SNPs was identified from successfully sequenced *P. vivax* isolates when compare to Sal‐I reference strain. The number of SNPs observed in this study was lower than those previously reported from India (85 SNPs), Korea (74 SNPs), and Myanmar (168 SNPs) but higher than that reported from Pakistan (34 SNPs) [17, 19–21]. The genetic analysis revealed low genetic diversity with a nucleotide diversity (*π*) of 0.00016 and haplotype diversity of 0.14904. Interestingly, despite the extremely low nucleotide diversity observed throughout the study period, temporal variation in haplotype distribution was evident. Only a single haplotype was detected in samples collected during 2017 and 2018, whereas six haplotypes were identified in 2019. This pattern may reflect the introduction of genetically distinct parasite variants or temporal changes in the local parasite population structure. In low‐transmission settings, parasite populations are often dominated by a limited number of clonal lineages, resulting in low overall genetic diversity [26]. However, sporadic introduction of new strains through human movement, ongoing local transmission, or the presence of hypnozoites may increase haplotype diversity without substantially altering overall nucleotide diversity [27]. The neutral evolutionary test, Tajima’s D value, was negative (Tajima’s D = −1.96374, *p* < 0.01), suggesting that the *vir* 12 gene may have been affected by population expansion [22]. Similarly, Fu and Li’s *D*∗ and F∗ value (−3.94143 and −3.88616, *p* < 0.02) provided evidence consistent with purifying selection of *vir* 12 gene. However, the observed patterns should be considered in the context of the very low nucleotide diversity of *vir 12* and the analysis of a single genetic locus. The negative values of the neutrality tests may reflect the combined effects of selection and demographic history, while the low polymorphism of *vir 12* may limit the resolution of these analyses. Thus, analyses incorporating multiple genetic loci or genome‐wide data would provide further insight into the evolutionary processes shaping *vir 12* diversity. These findings indicate that *vir* 12 exhibits low nucleotide diversity (*π* = 0.00016) in studied population, consistent with reports from India (*π* = 0.06401), Korea (*π* = 0.02027), Myanmar (*π* = 0.03508), and Pakistan (*π* = 0.01816) [18–21], though with an even lower value. In contrast, Tajima’s D suggested purifying selection in present dataset, whereas studies from Korea and Myanmar reported signals of balancing selection and/or decreasing population size.

The haplotype networks and genetic differentiation of 157 sequences from 5 Asian populations indicated geographical differentiation of *vir* 12 genes. Haplotypes were unique among geographically distant *P. vivax* isolates. The results indicate strong geographic differentiation among *P. vivax* populations, as evidenced by a high *F*_ST_ value.

This study provides the first insight into the genetic diversity and population characteristics of the *vir* 12 gene in Thai *P. vivax* isolates. However, this investigation is limited by the geographic sampling which was focused on a single province, the comparatively small number of gene targets analyzed and the unsuccessful amplification of 35% of collected samples, which were, therefore, excluded from subsequent sequencing and genetic analyses. Further studies should expand both the geographic scope and *vir* gene repertoire to allow comparative analyses across subfamilies. In addition, the development of alternative or newly designed primers targeting more conserved regions may improve amplification success, while analyses using additional genetic markers and larger sample sets could provide a more comprehensive understanding of *P. vivax* genetic diversity and population structure.

## 5. Conclusions

These findings indicate low genetic diversity of *vir* 12 among the successfully amplified *P. vivax* isolates from Yala province, Thailand, which differs from that reported in other endemic regions. These results highlight the potential importance of *vir* gene variation in immune evasion and malaria pathogenesis and support further investigations using larger sample sets and additional *vir* loci to better understand their epidemiological and functional significance.

## Author Contributions

Jiraporn Kuesap: conceptualization, methodology, formal analysis, data curation, supervision, writing–original draft, writing–review and editing, and funding acquisition. Nutnicha Suphakhonchuwong: methodology, formal analysis, and writing–review and editing.

## Funding

This research was supported by Thailand Science Research and Innovation (TSRI) Fundamental Fund, fiscal year 2024 [grant numbers TUFF63/2567].

## Disclosure

All authors read and approved the final manuscript.

## Conflicts of Interest

The authors declare no conflicts of interest.

## Supporting Information

Additional supporting information can be found online in the Supporting Information section.

## Supporting information


**Supporting Information** The global *vir* 12 sequences from India, Korea, Myanmar, Pakistan, and Thailand are provided as Table S1.

## Data Availability

The collected and analyzed data in this study are available from the corresponding author.
