# Supplementary material for: Exploring Genetic Polymorphism of Plasmodium vivax Variant Interspersed Repeats (vir) 12, Subfamily E, in Clinical Samples From Thailand
Source: J Trop Med. 2026 Oct 21;2026:5898346. doi: 10.1155/jotm/5898346 (PMC13685497; doi:10.1155/jotm/5898346)
Supplement: Supplementary file 1 [file JOTM-2026-5898346-s001.zip › Table S1 The global vir 12 sequences from India, Korea, Myanmar, Pakistan and Thailand_2.docx]

**Table S1:** The global *vir* 12 sequences from India, Korea, Myanmar, Pakistan and Thailand.

| **Country** | **n** | **GenBank Accession Numbers** |
| --- | --- | --- |
| India | 19 | JQ733953.1-JQ733971.1 |
| Korea | 56 | KY608364.1- KY608419.1 |
| Myanmar | 17 | MN436012.1- MN436028.1 |
| Pakistan | 6 | PP505464.1- PP505469.1 |
| Thailand, present study | 65 | PX696666-PX696730 |
